# Genomic Determinants of PI3K Pathway Inhibitor Response in Cancer

**DOI:** 10.3389/fonc.2012.00109

**Published:** 2012-08-31

**Authors:** Britta Weigelt, Julian Downward

**Affiliations:** ^1^Signal Transduction Laboratory, Cancer Research UK London Research InstituteLondon, UK; ^2^Division of Cancer Biology, The Institute of Cancer ResearchLondon, UK

**Keywords:** PI3K pathway inhibitors, drug response, genetic determinant, cancer

## Abstract

The phosphoinositide 3-kinase (PI3K) pathway is frequently activated in cancer as a result of genetic (e.g., amplifications, mutations, deletions) and epigenetic (e.g., methylation, regulation by non-coding RNAs) aberrations targeting its key components. Several lines of evidence demonstrate that tumors from different anatomical sites depend on the continued activation of this pathway for the maintenance of their malignant phenotype. The PI3K pathway therefore is an attractive candidate for therapeutic intervention, and inhibitors targeting different components of this pathway are in various stages of clinical development. Burgeoning data suggest that the genomic features of a given tumor determine its response to targeted small molecule inhibitors. Importantly, alterations of different components of the PI3K pathway may result in distinct types of dependencies and response to specific therapeutic agents. In this review, we will focus on the genomic determinants of response to PI3K, dual PI3K/mechanistic target of rapamycin (mTOR), mTOR, and AKT inhibitors in cancer identified in preclinical models and clinical trials to date, and the development of molecular tools for the stratification of cancer patients.

## Introduction

**Figure 1:**
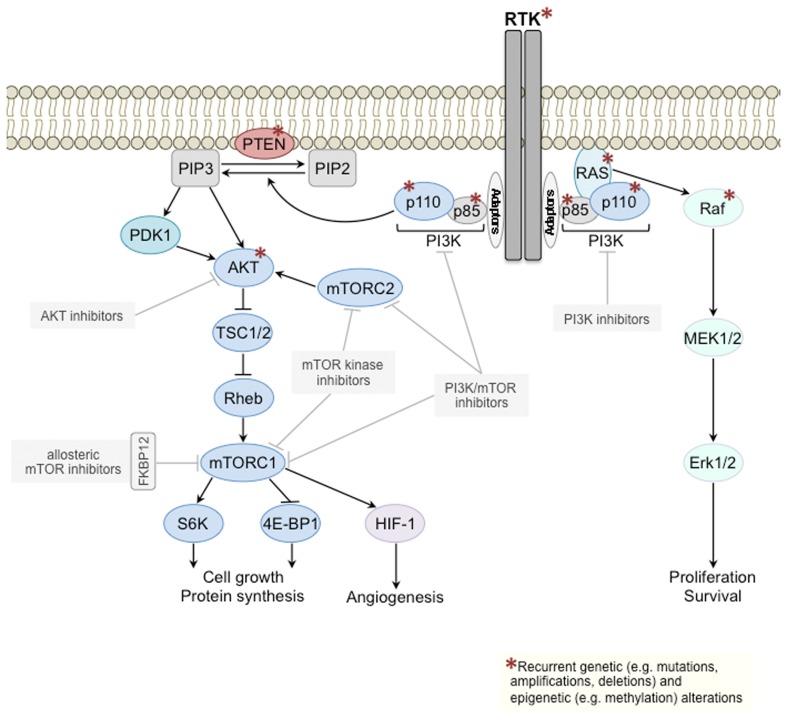
**Class I PI3K signal transduction pathway**. Components of the class I PI3K signaling pathway (left) and of the mitogen-activated protein kinase (MAPK) pathway (right) recurrently targeted by genetic/epigenetic alterations in cancer are depicted with a red asterisk. Several PI3K pathway inhibitors downstream of RTKs are currently being tested in clinical trials (gray boxes). mTOR, mechanistic target of rapamycin; mTORC, mTOR complex; PI3K, phosphoinositide 3-kinase; PIP2, phosphatidylinositol 4,5-bisphosphate; PIP3, phosphatidylinositol (3,4,5)-triphosphate; PTEN, phosphatase and tensin homolog; RTK, receptor tyrosine kinase; TSC, tuberous sclerosis protein.

The phosphoinositide 3-kinase (PI3K) signaling pathway regulates numerous processes in the normal cell such as growth, proliferation, survival, motility, and metabolism (Engelman et al., 2006). In human cancer, the PI3K pathway is one of the most frequently activated signal transduction pathways, and its prominent role is highlighted by the array of mechanisms targeting several of its key components (Figure 1). Mutations and/or amplifications of genes encoding receptor tyrosine kinases (RTKs) upstream of class I PI3Ks (glossary box), including the human epidermal growth factor receptors EGFR (*ERBB1*) and HER2 (*ERBB2*), of the PI3K catalytic subunits p110α (*PIK3CA*) and p110β (*PIK3CB*), the PI3K regulatory subunits p85α (*PIK3R1*) and p85β (*PIK3R2*), the PI3K effector AKT (*AKT1*), and of the PI3K activator KRAS are frequently observed in cancer [Catalog Of Somatic Mutations In Cancer (COSMIC), http://www.sanger.ac.uk/cosmic; Forbes et al., 2011], as is loss of function of the tumor suppressors phosphatase and tensin homolog (PTEN) and inositol polyphosphate 4-phosphatase-II (INPP4B), negative regulators of PI3K signaling, through mutations, deletions, or epigenetic mechanisms (Gewinner et al., 2009; Fedele et al., 2010; Hollander et al., 2011).

Given that the PI3K pathway is frequently activated in cancers, that tumorigenesis and/or maintenance of the malignant phenotype of different tumor types is driven by its continued activation (Bader et al., 2005; Hollander et al., 2011), and that kinases are amendable to pharmacological intervention, it is not surprising that there has been great interest in the development of allosteric and ATP-competitive small molecule inhibitors targeting different components of this pathway downstream of RTKs (Liu et al., 2009). These targeted agents include PI3K inhibitors, either isoform specific [i.e., class I isoforms p110α, p110β, p110γ, p110δ; (glossary box)] or pan-class I PI3K inhibitors, dual PI3K/mechanistic target of rapamycin (mTOR) inhibitors, mTOR inhibitors, and AKT inhibitors, which are all currently in various stages of clinical development (Table 1).

**Table 1:** Open clinical trials testing PI3K pathway inhibitors in cancer*.

Inhibitor name	Company	Target	Clinical trial phase	Cancer type
**PAN-CLASS I PI3K INHIBITORS**				
BAY80-6946	Bayer	Class I PI3K	I	Advanced solid cancers
ZSTK474	Zenyaku Kogyo	Class I PI3K	I	Advanced solid cancers
GSK1059615	GlaxoSmithKline	Class I PI3K	I	Terminated
BKM120	Novartis	Class I PI3K	I and II	(Advanced) solid cancers; NSCLC, endometrial, prostate, breast, colorectal, pancreatic, renal cell, GIST, melanoma, glioblastoma, leukemia, SCCHN, TCC
GDC-0941	Roche/Genentech	Class I PI3K	I and II	Solid cancers; breast, NSCLC, non-Hodgkin’s lymphoma
PX866	Oncothyreon	Class I PI3K	I and II	Prostate, NSCLC, SCCHN, colorectal, glioblastoma
XL147 (SAR245408)	Exelixis/Sanofi-Aventis	Class I PI3K	I and II	Solid cancers; endometrial, ovarian, breast, NSCLC
**ISOFORM SPECIFIC PI3K INHIBITORS**				
BYL719	Novartis	p110α	I	Advanced solid cancers; SCCHN
GDC-0032	Roche/Genentech	p110α	I	Solid cancers
INK-1117	Intellikine	p110α	I	Advanced solid cancers
GSK2636771	GlaxoSmithKline	p110β	I/IIa	Advanced solid cancers (PTEN deficient)
IPI-145	Infinity	p110γ, p110δ	I	Advanced hematological malignancies
AMG319	Amgen	p110δ	I	Relapsed or refractory lymphoid malignancies
CAL-101 (GS-1101)	Gilead sciences	p110δ	I, II, and III	Chronic lymphocytic leukemia, Hodgkin lymphoma, non-Hodgkin’s lymphoma; mantle cell lymphoma, acute myeloid leukemia, multiple myeloma
**DUAL PI3K/mTOR INHIBITORS**				
DS-7423	Daiichi Sankyo	PI3K/mTOR	I	Advanced solid cancers; colorectal, endometrial
GDC-0980	Roche/Genentech	PI3K/mTOR	I	(Advanced) solid cancers; non-Hodgkin’s lymphoma, breast, prostate, endometrial, renal cell
GSK2126458	GlaxoSmithKline	PI3K/mTOR	I	Advanced solid cancers
PWT33597	Pathway Therapeutics	PI3K/mTOR	I	Advanced solid cancers or malignant lymphoma
SF1126	Semafore	PI3K/mTOR	I	Advanced solid cancers
BEZ235	Novartis	PI3K/mTOR	I and II	Advanced solid cancers; renal cell, breast
BGT226	Novartis	PI3K/mTOR	I and II	Completed (advanced solid cancers; breast)
PF-04691502	Pfizer	PI3K/mTOR	I and II	Advanced solid cancers; breast, endometrial
PF-05212384 (PKI-587)	Pfizer	PI3K/mTOR	I and II	Advanced solid cancers; endometrial
XL765 (SAR245409)	Exelixis/Sanofi-Aventis	PI3K/mTOR	I and II	Advanced breast, gliomas, glioblastoma multiforme
**mTOR KINASE INHIBITORS**				
AZD2014	AstraZeneca	mTOR	I	Advanced solid cancers; breast
AZD8055	AstraZeneca	mTOR	I	Recurrent glioma
INK-128	Intellikine	mTOR	I	Advanced solid cancers; multiple myeloma, Waldenstrom macroglobulinemia
OSI-027	Astellas Pharma	mTOR	I	Advanced solid cancers; lymphoma
CC-223	Celgene Corporation	mTOR	I and II	Advanced solid cancers; non-Hodgkin’s lymphoma, multiple myeloma, NSCLC
**ALLOSTERIC mTOR INHIBITORS (RAPAMYCIN ANALOGS)**				
Sirolimus (Rapamycin)	Wyeth/Pfizer	mTOR	I, II, and III	Advanced solid cancers; breast, liver, rectum, NSCLC, leukemias, lymphomas, head and neck, pancreatic, ovarian, fallopian tube, glioblastoma, fibromatosis
Everolimus** (RAD001)	Novartis	mTOR	I, II, and III	Solid cancers; leukemias, lymphomas, breast, bladder, head and neck, kidney/renal cell, liver, gastric, thyroid, neuroendocrine tumors, ovarian, fallopian tube, cervical, colorectal, brain and central nervous system, prostate, endometrial, esophageal, melanoma, NSCLC, SCLC, germ cell, soft tissue sarcoma, osteosarcoma, nasopharyngeal, glioma, Waldenstrom’s macroglobulinemia
Temsirolimus** (CCI-779)	Wyeth/Pfizer	mTOR	I and II	Advanced solid cancers; breast, endometrial, ovarian, prostate, liver, kidney/renal cell, SCCHN, NSCLC, melanoma, sarcoma, lymphomas, leukemia, brain and central nervous system, bladder, urethral
Ridaforolimus (MK-8669)	Merck/Ariad	mTOR	I	Advanced solid cancers; endometrial, ovarian, breast, NSCLC, renal cell, soft tissue sarcoma
**AKT INHIBITORS (ATP-Competitive)**				
ARQ 092	ArQule/Daiichi Sankyo	AKT	I	Advanced solid cancers
AZD5363	AstraZeneca	AKT	I	Advanced solid cancers
GSK2141795	GlaxoSmithKline	AKT	I	Completed/not recruiting (advanced solid cancers; lymphoma)
GDC-0068	Roche/Genentech	AKT	I and II	Advanced solid cancers; prostate cancer
GSK2110183	GlaxoSmithKline	AKT	I and II	Solid cancers, hematological malignancies, multiple myeloma, Langerhans cell histiocytosis, chronic lymphocytic leukemia
**ALLOSTERIC AKT INHIBITORS**				
MK-2206	Merck	AKT	I and II	Advances solid cancers; breast, endometrial, ovarian, fallopian tube, peritoneal, gastric, gastroesophageal junction, colorectal, prostate, NSCLC, SCLC, melanoma, kidney, leukemias, lymphomas, biliary, head and neck, liver, thymic, nasopharyngeal

*Data retrieved from http://clinicaltrials.gov and http://www.fda.gov/ (May 2012).

**Temsirolimus: approved for the treatment of advanced renal cell carcinoma; Everolimus: approved for the treatment of progressive neuroendocrine tumors of pancreatic origin, for advanced renal cell carcinoma after failure of treatment with sunitinib or sorafenib, for renal angiomyolipoma and tuberous sclerosis complex, and for subependymal giant cell astrocytoma associated with tuberous sclerosis.

*GIST, gastrointestinal stromal tumor; NSCLC, non-small cell lung cancer; SCCHN, squamous cell carcinoma of the head and neck; SCLC, small cell lung cancer; TCC, transitional cell carcinoma of the urothelium*.

Over the past years it has become apparent that irrespective of the cancer type and small molecule inhibitor or antibody used, kinase inhibitor response is limited to those tumors whose proliferation and survival are reliant on the activation of the targeted oncogenic kinase (Sharma and Settleman, 2007; Janne et al., 2009). Bernard Weinstein coined the term “oncogene addiction” to describe this phenomenon (Weinstein, 2002), which has important implications for the targeting of kinases: given the incredibly diverse repertoire of genetic and epigenetic aberrations observed within a given cancer type, only the subset of tumors “addicted” to the continued activation of the oncogenic kinase targeted will prove vulnerable to the therapeutic intervention. Consistent with this “oncogene addiction” concept, strong associations between a tumor’s genotype and its response to small molecule kinase inhibitors or antibodies targeting kinases have been identified. For example, melanomas harboring *BRAF*^V600E^ mutations are selectively sensitive to the BRAF inhibitor Vemurafenib (Flaherty et al., 2010), non-small cell lung cancers (NSCLCs) harboring *EGFR* mutations to the EGFR inhibitors Gefitinib or Erlotinib (Pallis et al., 2011), *HER2* amplified breast and gastric cancers to the HER2 targeting agents Trastuzumab or Lapatinib (Stern, 2012), and *KIT* and *PDGFRA* mutant gastrointestinal stromal tumors to Imanitib Mesylate and other small molecule inhibitors targeting mutant KIT and PDGFRα (Antonescu, 2011). Importantly, however, cancers harboring only wild-type copies of the genes mentioned above seem not to be sensitive to the same agents.

As PI3K pathway inhibitors progress into trials focusing on their clinical efficacy (Table 1), it is critical to identify their genomic determinants of response and to select the patient population most likely to benefit from treatment. In fact, it has been suggested to incorporate predictive biomarkers throughout the clinical drug development process from phase I studies onward in order to enrich trials with patients more likely to respond to a given targeted therapy and to increase the chances of drug registration (Carden et al., 2010). For the guidance and prioritization of predictive biomarker candidates in early clinical trials, results derived from the study of preclinical models are of importance.

In this review, we focus on the genomic determinants of response to PI3K pathway inhibitors in cancer identified in preclinical models and clinical trials to date, and discuss the challenges for the development of molecular tools for the stratification of cancer patients.

## Genomic Determinants of PI3K Pathway Inhibitor Response in Preclinical Models

The ease of therapeutic intervention using *in vitro* cell culture and the wealth of data available on the mutational landscape of known cancer genes in the most common cell lines obtainable from commercial repositories have made cancer cell line panels the model of choice for the preclinical study of drug response. Furthermore, with the advent of methods for massively parallel sequencing, it is now possible to identify the genomic determinants of therapy response in *in vitro* models in a genome-wide fashion (Barretina et al., 2012; Garnett et al., 2012). In general, sensitivity or resistance of cancer cell lines to a given targeted agent are determined by short-term treatment ranging from 48 to 120 h of cells grown on tissue culture plastic using several dilutions of the inhibitor. At the endpoint, cell number or cell viability is assessed and drug response reported as half-maximal inhibitory concentration (IC_50_), or the concentration needed to reduce the growth of treated cells to half that of untreated or vehicle treated cells (GI_50_). In addition, xenograft studies in immunodeficient mice injected with human cancer cell lines or human tumor tissues, as well as transgenic mouse models have been employed to assess anti-tumor activity of PI3K pathway inhibitors *in vivo* using tumor growth, proliferation, apoptosis, and/or levels of pathway activation state as read-out of treatment response.

Using these preclinical approaches, several groups attempted to define genomic determinants of response to PI3K pathway inhibitors. It should perhaps not come as a surprise that genetic alterations leading to PI3K pathway activation, including *PIK3CA* gain-of-function mutations and/or *PTEN* mutations/PTEN loss of function and/or amplification of *HER2*, have been repeatedly identified as predictors of response to these agents (Table 2). However, tumor type-specific differences have been observed. For example, in ovarian cancer cells both *PIK3CA* mutations and PTEN deficiency have been reported to predict PI3K pathway inhibitor response (Ihle et al., 2009; Di Nicolantonio et al., 2010; Meuillet et al., 2010; Santiskulvong et al., 2011; Tanaka et al., 2011; Meric-Bernstam et al., 2012), whereas in breast cancer the associations between PTEN loss of function and response are less clear (She et al., 2008; Brachmann et al., 2009; Lehmann et al., 2011; Sanchez et al., 2011; Tanaka et al., 2011; Weigelt et al., 2011), which will be discussed in greater detail below.

**Table 2:** **Genomic determinants of response to PI3K pathway inhibitors identified in preclinical cancer models**.

Inhibitor (target)	Cancer type	Preclinical model	Genomic determinant of response	Reference
GDC-0941 (Class I PI3K)	Breast	Cell lines	*PIK3CA* mutation	O’Brien et al. (2010)
		Cell line xenografts	*HER2* amplification	
BEZ235 (PI3K/mTOR)	Breast	Cell lines	*PIK3CA* mutation	Serra et al. (2008)
		Cell line xenografts	
BEZ235 (PI3K/mTOR)	Breast	Cell lines	*PIK3CA* mutation	Brachmann et al. (2009)
		Cell line xenografts	*HER2* amplification	
BEZ235 (PI3K/mTOR)	Breast	Cell lines	*PIK3CA* mutation (PTEN deficiency)	Lehmann et al. (2011)
BKM120 (Class I PI3K), BGT226 (PI3K/mTOR), Everolimus (mTOR)	Breast	Cell lines	*PIK3CA* mutation	Sanchez et al. (2011)
PP242 (mTOR kinase)	Breast	Cell lines	*PIK3CA* mutation	Weigelt et al. (2011)
Everolimus (mTOR)			*HER2* amplification (only for PP242)	
Rapamycin (mTOR)	Breast	Cell lines	None (not *PIK3CA* mutations)	Loi et al. (2010)
AKTi-1/2 (AKT)	Breast	Cell lines	*PIK3CA* mutation	She et al. (2008)
		Cell line xenografts	*HER2* amplification	
Everolimus (mTOR)	Non-malignant breast	Cell lines (isogenic)	*PIK3CA* mutation (knock-in)	Di Nicolantonio et al. (2010)
Temsirolimus (mTOR)	Multiple myeloma	Cell lines	PTEN deficiency	Shi et al. (2002)
Everolimus (mTOR)	Glioblastoma multiforme	Cell lines Human tumor xenografts	None (not PTEN deficiency)	Yang et al. (2008)
BEZ235 (PI3K/mTOR)	Ovarian	Cell lines	*PIK3CA* mutation	Santiskulvong et al. (2011)
			PTEN deficiency	
WAY-175, WAY-176 (Class I PI3K)	Various (breast, prostate, melanoma, lung, colon)	Cell lines	*PIK3CA* mutation	Yu et al. (2008)
PX866 (PI3K)	Various (non-small cell lung cancer, colon, breast, pancreatic, prostate, ovarian, multiple myeloma)	Cell line xenografts	*PIK3CA* mutation PTEN deficiency	Ihle et al. (2009)
CH5132799 (PI3K)	Various (breast, ovarian, prostate, endometrial)	Cell lines Cell line xenografts	*PIK3CA* mutation	Tanaka et al. (2011)
Temsirolimus (mTOR)	Various (glioblastoma, prostate)	Cell lines	PTEN deficiency	Neshat et al. (2001)
Everolimus (mTOR)	Various (prostate, glioblastoma, breast, ovarian, cervical)	Cell lines	*PIK3CA* mutation PTEN deficiency	Di Nicolantonio et al. (2010)
Rapamycin (mTOR)	Various (neuroendocrine, cervical, hepatocellular, melanoma, ovarian, colon, breast, renal cell, glioblastoma, breast)	Cell lines	*PIK3CA* mutation PTEN deficiency	Meric-Bernstam et al. (2012)
PHT-427 (AKT/PDPK1)	Various (pancreatic, prostate, ovarian, breast, lung)	Cell line xenografts	*PIK3CA* mutation	Meuillet et al. (2010)
25 PI3K pathway inhibitors (PI3K, PI3K/mTOR, AKT)	Various (lung, colorectal, gastric, breast, ovarian, brain, renal, melanoma, prostate)	Cell lines	None (p-AKT levels)	Dan et al. (2010)
A-443654 (AKT)	Various (bladder, blood, bone, breast, CNS, GI tract, kidney, lung, ovary, pancreas, skin, soft tissue, thyroid, upper aerodigestive, uterus)	Cell lines	*SMAD4* mutation	Garnett et al. (2012); (http://www.cancerrxgene.org/; Release 2, July 2012)
AKT inhibitor VIII (AKT)	Various (bladder, blood, bone, breast, CNS, GI tract, kidney, liver, lung, ovary, pancreas, prostate, skin, soft tissue, thyroid, upper aerodigestive, uterus)	Cell lines	*PIK3CA* mutation *ERBB2* mutation	Garnett et al. (2012); (http://www.cancerrxgene.org/; Release 2, July 2012)
MK-2206 (AKT)	Various (bladder, blood, bone, breast, CNS, GI tract, kidney, liver, lung, ovary, pancreas, prostate, skin, soft tissue, thyroid, upper aerodigestive, uterus)	Cell lines	*PTEN* mutation	Garnett et al. (2012); (http://www.cancerrxgene.org/; Release 2, July 2012)
AZD6482 (p110β)	Various (bladder, blood, bone, breast, CNS, GI tract, kidney, liver, lung, ovary, pancreas, prostate, skin, soft tissue, thyroid, upper aerodigestive, uterus)	Cell lines	*PTEN* mutation *PIK3CA* mutation	Garnett et al. (2012); (http://www.cancerrxgene.org/; Release 2, July 2012)
BEZ235 (PI3K/mTOR)	Various (bladder, blood, bone, breast, CNS, GI tract, kidney, liver, lung, ovary, pancreas, prostate, skin, soft tissue, thyroid, upper aerodigestive, uterus)	Cell lines	*CDKN2A* mutation *NRAS* mutation	Garnett et al. (2012); (http://www.cancerrxgene.org/; Release 2, July 2012)
Temsirolimus (mTOR)	Various (bladder, blood, bone, breast, CNS, GI tract, kidney, liver, lung, ovary, pancreas, prostate, skin, soft tissue, thyroid, upper aerodigestive, uterus)	Cell lines	*PTEN* mutation	Garnett et al. (2012); (http://www.cancerrxgene.org/; Release 2, July 2012)
GDC-0941 (Class I PI3K), AZD8055 (mTOR kinase), Rapamycin (mTOR), JW-7-52-1 (mTOR)	Various (bladder, bone, breast, CNS, GI tract, kidney, liver, lung, ovary, pancreas, prostate, skin, soft tissue, thyroid, upper aerodigestive, uterus)	Cell lines	None (*TET2* mutations associated with AZD8055 response, however only 3/554 cell lines were *TET2* mutant)	Garnett et al. (2012); (http://www.cancerrxgene.org/; Release 2, July 2012)
**CONFIRMATORY STUDIES USING ANIMAL MODELS**				
BEZ235 (PI3K/mTOR)	Prostate and glioblastoma	Cell line xenografts	PTEN deficiency	Maira et al. (2008)
Rapamycin (mTOR)	Breast and pancreatic	Cell line xenografts	*PIK3CA* mutation	Meric-Bernstam et al. (2012)
WYE-354 (mTOR kinase)	Prostate and glioblastoma	Cell line xenografts	PTEN deficiency	Yu et al. (2009)
BEZ235 (PI3K/mTOR)	Lung	*PIK3CA* H1047R mouse model	*PIK3CA* H1047R mutation	Engelman et al. (2008)
Rapamycin (mTOR), API-2 (AKT)	Ovarian endometrioid adenocarcinoma	*Apc*^flox/flox^; *Pten*^flox/flox^ mouse model	PTEN deficiency	Wu et al. (2011)

*CNS, central nervous system; GI, gastrointestinal*.

Several studies provided evidence to suggest that cancer cells harboring *PIK3CA* gain-of-function mutations are selectively sensitive to inhibitors of different components of the PI3K pathway. In breast cancer, cell culture, and/or xenograft models identified *PIK3CA* mutations as determinant of response to PI3K inhibition (O’Brien et al., 2010; Sanchez et al., 2011), dual PI3K/mTOR inhibition (Serra et al., 2008; Brachmann et al., 2009; Lehmann et al., 2011; Sanchez et al., 2011), mTOR kinase inhibition (Weigelt et al., 2011), allosteric mTOR inhibition (Sanchez et al., 2011; Weigelt et al., 2011), and AKT inhibition (She et al., 2008; Meuillet et al., 2010; Table 2). In one report, however, which assessed seven estrogen receptor (ER)-positive breast cancer cell lines and their response to the allosteric mTOR inhibitor Rapamycin (Sirolimus), no correlation with *PIK3CA* mutation status but to some extent with a *PIK3CA* mutation associated gene signature was found (Loi et al., 2010). *In vitro* and xenograft models of breast cancer have also demonstrated that cells harboring amplification of the RTK *HER2* are dependent on PI3K pathway activation and sensitive to its inhibition through targeting of PI3K (O’Brien et al., 2010; Tanaka et al., 2011), dual PI3K/mTOR (Brachmann et al., 2009), AKT (She et al., 2008), and mTOR kinase (Weigelt et al., 2011). In fact, mTOR kinase inhibitors seem to lead to a more effective decrease of PI3K pathway signaling than allosteric mTOR inhibitors given that *HER2* amplified breast cancer cells *in vitro* have been found to be unresponsive to the rapamycin analog (“rapalog”) Everolimus (RAD001; glossary box; Weigelt et al., 2011). Whereas *PIK3CA* mutations and *HER2* amplification have been identified in the majority of preclinical breast cancer studies as determinant of sensitivity to PI3K pathway inhibition downstream of RTKs, the correlation between PTEN deficiency and response is less clear. In some studies, results were inconclusive as only a subset of PTEN null breast cancer cell lines were sensitive to PI3K pathway inhibition (She et al., 2008; Lehmann et al., 2011; Sanchez et al., 2011), whilst others found PTEN deficient breast cancer cells to be preferentially resistant to treatment with PI3K (Tanaka et al., 2011), dual PI3K/mTOR (Brachmann et al., 2009), mTOR kinase, and allosteric mTOR inhibitors (Weigelt et al., 2011). These data are consistent with the notion that aberrations in the different components of the PI3K pathway are not necessarily equivalent in their biological impact and their potential to activate the signaling pathway (Stemke-Hale et al., 2008; Vasudevan et al., 2009; Dan et al., 2010). Moreover, these observations also suggest that sensitivity of PTEN deficient breast cancer cells to PI3K pathway inhibitors may be dependent on epistatic interactions between PI3K pathway genes and genes from other signaling pathways such as the MAPK pathway, as well as the release of negative feedback loops and the node targeted by pharmacologic inhibition (Efeyan and Sabatini, 2010; Zhang and Yu, 2010). Recent work in preclinical models has suggested that PTEN deficient cancers may depend on p110β rather than p110α signaling (Jia et al., 2008; Wee et al., 2008; Edgar et al., 2010; Ni et al., 2012), and a p110β isoform specific inhibitor (GSK2636771) is currently being tested in a clinical trial of PTEN deficient malignancies (NCT01458067). In fact, as in different disease contexts selective targeting of specific p110 isoforms may be more beneficial and less toxic than pan-PI3K inhibition (Jia et al., 2009; Vanhaesebroeck et al., 2010; Jamieson et al., 2011; Tzenaki et al., 2012), also p110α, p110γ, and p110δ specific inhibitors are being assessed in clinical trials (Table 1). The contribution of the p85 isoforms (glossary box) to PI3K inhibitor response is however not yet fully understood. There is evidence to suggest that different cancer types express different levels of p110 and p85 isoforms (Cortes et al., 2012; Tzenaki et al., 2012), which may lead to tumor type-specific combinations of catalytic and regulatory PI3K subunits. It remains to be determined whether certain PI3K inhibitors show preferential activity against specific p110/p85 isoform combinations and whether distinct mutations in the regulatory subunits *PIK3R1* or *PIK3R2* have an impact on PI3K inhibitor response.

The general effect of *PIK3CA* gain-of-function mutations in the sensitization to PI3K pathway inhibitors has been confirmed in a mouse model with inducible expression of human oncogenic p110α (i.e., p110α H1047R), where treatment of the p110α H1047R driven lung adenocarcinomas with the dual PI3K/mTOR inhibitor BEZ235 led to marked tumor regression (Engelman et al., 2008). In xenografts derived from the breast cancer cell line MCF7 and the pancreatic carcinoid cell line BON, both harboring an activating *PIK3CA* mutation, treatment with the allosteric mTOR inhibitor Rapamycin (Sirolimus) was associated with a significant decrease in tumor volume (Meric-Bernstam et al., 2012). Moreover, using *PIK3CA* wild-type human breast immortalized epithelial cells (hTERT-HME1) or non-malignant MCF10A breast cells, knock-in of the E454K or H1047R *PIK3CA* mutant alleles sensitized non-transformed human breast cells to the rapalog Everolimus (Di Nicolantonio et al., 2010).

Also when focusing on an array of tumor types rather than on a single disease entity, *PIK3CA* mutant cell lines, or cell line derived xenografts were found to be selectively sensitive to PI3K pathway inhibition (Table 2). For example, analysis of xenografts derived from pancreatic, prostate, ovarian, NSCLC, and ovarian cancer cells revealed that those harboring *PIK3CA* mutations were among the most sensitive to the AKT inhibitor PHT-427 (Meuillet et al., 2010). This observation has been validated in a large panel of breast, ovarian, prostate, and endometrial cancer cells, given that those with *PIK3CA* mutations were found to be significantly more sensitive to the PI3K inhibitor CH5132799 *in vitro* than those without (Tanaka et al., 2011). Other studies assessing mixed tumor type cell line panels, however, have identified both activating *PIK3CA* mutations and PTEN loss of function as determinant of PI3K pathway inhibitor response. This was observed in a panel of breast, melanoma, lung, colon, prostate cancer cells treated *in vitro* with the PI3K inhibitors WAY-175 and WAY-176 (Yu et al., 2008), in a panel of human lung, colon, breast, pancreatic, ovarian, and multiple myeloma cell line derived xenografts treated with the PI3K inhibitor PX866 (Ihle et al., 2009), and in cell line panels of various tumor types treated *in vitro* with the allosteric mTOR inhibitors Everolimus (Di Nicolantonio et al., 2010) or Rapamycin (Meric-Bernstam et al., 2012). In one study, the evaluation of the *in vitro* efficacy of 25 PI3K pathway inhibitors in a panel of 39 human cancer cell lines did not identify any genetic determinant of sensitivity (Dan et al., 2010).

It is interesting to note that whilst loss of PTEN function has been shown to be a strong activator of the PI3K pathway as determined by levels of AKT phosphorylation (Stemke-Hale et al., 2008), only a few studies identified *PTEN* mutations/PTEN deficiency as a single genomic determinant of response to PI3K pathway inhibitors. Murine PTEN deficient ovarian endometrioid adenocarcinomas arising in *Apc^flox/flox^*; *Pten^flox/flox^* mice have been shown to be sensitive to Rapamycin and the AKT inhibitor API-2 (Wu et al., 2011). Also in a panel of multiple myeloma (Shi et al., 2002), glioblastoma, and prostate cancer cell lines (Neshat et al., 2001), PTEN deficiency was reported to be associated with enhanced sensitivity to the allosteric mTOR inhibitor Temsirolimus (CCI-779). Consistent with this result, the PTEN null glioblastoma cell line U-87MG and the prostate cancer cell line PC3 were found to be sensitive to Rapamycin *in vitro* (Di Nicolantonio et al., 2010), and when grown as xenografts to the dual PI3K/mTOR inhibitor BEZ235 (Maira et al., 2008) and the ATP-competitive mTOR inhibitor WYE-354 (Yu et al., 2009). At variance with these findings, PTEN loss of function was shown to be a poor predictor of Everolimus response in a panel of 17 glioblastoma multiforme cell lines, and in human glioblastoma xenograft models (Yang et al., 2008).

The data discussed above on the genomic determinants of PI3K pathway inhibitor response identified *in vitro* are based on the analysis of up to 60 cancer cell lines, which were selected based on different criteria by independent investigators. Recently, two large-scale studies subjected hundreds of cancer cell lines derived from tumors stemming from different anatomical sites and tissue types to transcriptomic profiling, copy number profiling, and massively parallel sequencing. Owing to their unprecedented scale and approach employed, these studies unraveled several associations between genetic aberrations and response to specific targeted therapies (Barretina et al., 2012; Garnett et al., 2012). Garnett et al. (2012) tested up to 714 cell lines for their response to 138 anticancer agents including ten PI3K pathway inhibitors downstream of RTKs (http://www.cancerrxgene.org/; Release 2, July 2012), and observed that, in line with previous findings, cancer cells harboring mutations in *PIK3CA* and *PTEN* were sensitive to treatment with the AKT inhibitor VIII and MK-2206, respectively. Of note, also *ERBB2* mutations were associated with AKT inhibitor VIII response. Sensitivity to the AKT inhibitor A-443654 and the dual PI3K/mTOR inhibitor BEZ235, however, was not determined by PI3K pathway aberrations but by the presence of *SMAD4* and *CDKN2A* mutations, respectively (Table 2). In Garnett et al. (2012) mutations in *PTEN* were associated with response to the mTOR inhibitor Temsirolimus, and not only *PTEN* but also *PIK3CA* mutations predicted response to the PI3K isoform specific p110β inhibitor AZD6482 (http://www.cancerrxgene.org/; Release 2). On the other hand, contrary to previous reports, no mutations predictive of response to the PI3K inhibitor GDC-0941, the mTOR kinase inhibitor AZD8055, and the mTOR inhibitors Rapamycin and JW-7-52-1 were identified (http://www.cancerrxgene.org/; Release 2; Garnett et al., 2012; Table 2).

Mutation analysis has already become part of the diagnostic armamentarium for lung and colon cancers (Allegra et al., 2009; Keedy et al., 2011), and is also likely to be implemented in the management of other tumor types. In fact, the potential determinants of PI3K pathway inhibitor response identified in preclinical studies may provide a rationale for the guidance of predictive biomarkers to be assessed in early clinical trials. It should be noted here that in addition to genomic response predictors also non-genetic predictors of PI3K inhibitor response have been put forward, yet none of them has been fully validated. In breast cancer, a gene expression signature predictive of *in vitro* sensitivity to the PI3K inhibitor GDC-0941 (O’Brien et al., 2010), and a *PIK3CA* mutation associated gene signature (*PIK3CA*-GS) derived from exon 20 *PIK3CA* mutations able to predict *PIK3CA* mutation status in primary breast cancers and predictive of Rapamycin response *in vitro* have recently been described (Loi et al., 2010). In addition, several groups found increased phosphorylated (p)-AKT baseline levels as a read-out for PI3K pathway activation to be associated with its therapeutic intervention (Noh et al., 2004; Yu et al., 2008; Dan et al., 2010; Meric-Bernstam et al., 2012). Despite the potential utility of these approaches, it should be mentioned that gene expression signatures and immunohistochemical assessment of phosphorylated proteins have proven challenging to implement in routine clinical practice (Pinhel et al., 2010; Weigelt et al., 2012).

Although predictive markers of sensitivity to PI3K pathway inhibitors, such as *PIK3CA* mutations, are of importance for treatment tailoring, markers predictive of resistance may be useful. In fact, tumors harboring a given therapeutic target not uncommonly display primary (i.e., *de novo*) resistance or develop resistance over time (van der Heijden and Bernards, 2010; Turner and Reis-Filho, 2012). In several studies discussed here assessing determinants of single agent PI3K pathway inhibitor response, *KRAS* mutations were found to be associated with resistance to these targeted agents (Engelman et al., 2008; Brachmann et al., 2009; Ihle et al., 2009; Dan et al., 2010; Meuillet et al., 2010; Garnett et al., 2012), as were mutations in *APC*, *BRAF*, or *MYCN* (http://www.cancerrxgene.org/; Garnett et al., 2012).

Finally, in addition to genetic alterations of components of the PI3K pathway germline polymorphisms may affect response of patients treated with targeted therapies. Ng et al. (2012) have recently identified a common intronic deletion polymorphism of the *BIM* gene that leads to the generation of an alternative spliced BIM isoform lacking the BH3 domain, which is required for tyrosine kinase inhibitor induced apoptosis. This polymorphism was shown to confer intrinsic resistance to RTK inhibitors in chronic myeloid leukemia and *EGFR* mutated NSCLC cell lines (Ng et al., 2012). It is plausible that this and other germline polymorphisms may results in resistance to agents targeting the PI3K pathway.

Taken together, preclinical studies focusing on breast cancer only have repeatedly identified *PIK3CA* mutations and *HER2* amplifications as predictors of sensitivity to PI3K pathway inhibitors. In other tumor types, however, the genotype-drug response associations are less defined and *PIK3CA* mutations, PTEN loss of function or both, or *CDKN2A* mutations have been reported as determinants of response. Furthermore, the *in vitro* and animal model studies revealed that in cancer cells other than breast cancer, where MAPK pathway mutations are rare (COSMIC), *KRAS* mutations may confer resistance to single agent PI3K pathway inhibitor treatment, as do mutations in *BRAF*, *APC* and *MYCN*.

## Genomic Determinants of PI3K Pathway Inhibitor Response in Clinical Trials

Rapamycin analogs (“rapalogs”; glossary box) were the first PI3K pathway inhibitors to be tested in clinical trials for the treatment of cancer, and Everolimus and Temsirolimus have been approved by the US Food and Drug Administration (FDA) for the treatment of advanced renal cell carcinoma (ARCC), and Everolimus has also been approved for the treatment of progressive neuroendocrine tumors of pancreatic origin and non-malignant kidney and brain tumors (Table 1).

The determinants of mTOR inhibition in renal cell carcinomas may differ from those of other solid malignancies. In fact, clear cell renal cell carcinomas rarely harbor mutations in PI3K pathway components (COSMIC), however commonly show loss of function of the tumor suppressor genes PTEN (Brenner et al., 2002; Velickovic et al., 2002) or von Hippel Lindau (VHL), a critical regulator of the hypoxic response (Kim and Kaelin, 2004; Linehan et al., 2010). Clear cell renal cell cancer has been suggested to be a cell metabolism, angiogenesis-dependent and hypoxia-driven disease, and its response to mTOR inhibition thought to stem from its impact on proliferation and cell survival but also from the fact that the hypoxia-inducible-factor 1-α (HIF1-α) is under translational control of the mTOR complex 1 (mTORC1; glossary box; Thomas et al., 2006; Linehan et al., 2010). Exploratory subgroup analysis of the 209 patients from the Temsirolimus single agent arm of the phase III global ARCC trial (Hudes et al., 2007) investigated PTEN and HIF1-α protein expression levels by immunohistochemistry (IHC) on formalin fixed paraffin embedded nephrectomy or core biopsy derived tissues. Importantly, baseline PTEN or HIF1-α levels were shown not to be associated with single agent Temsirolimus response (Figlin et al., 2009; Table 3). Furthermore, in a retrospective subgroup analysis from a phase II clinical trial of ARCC (Atkins et al., 2004) including 20 patients (Cho et al., 2007), carbonic anhydrase IX (CA9), p-AKT, and PTEN protein expression levels using IHC or *VHL* mutation status were shown not to be significantly associated with single agent Temsirolimus response. There was, however, a significant positive association between higher p-rpS6 expression, a downstream effector of mTORC1 (Figure 1), and clinical Temsirolimus response (Cho et al., 2007). It should be noted that the analysis above was performed in a limited number of patients and their statistical power to reveal the associations should be taken into account.

**Table 3:** **Genomic determinants of response to PI3K pathway inhibitors identified in clinical trials**.

Inhibitor name (target)	Cancer type	Clinical trial	Patients (n)	Genomic determinant of sensitivity	Non-genomic determinant of sensitivity	Genomic determinant of resistance	Reference
Temsirolimus (mTOR), single agent	Renal cell carcinoma	NCT00065468 (retrospective subgroup analysis; phase III)	209 (Temsirolimus arm; ∼60% assessed)	NA	None (PTEN or HIF1-α protein expression assessed)	NA	Figlin et al. (2009)
Temsirolimus (mTOR), single agent	Renal cell carcinoma	ND (retrospective subgroup analysis; phase II)	20	None (VHL mutation assessed)	p-rpS6 (Ser235)	NA	Cho et al. (2007)
Temsirolimus (mTOR), single agent, or combined; Rapamycin (mTOR), combined; PX866 (PI3K), single agent	Breast, cervical, endometrial, ovarian cancer (colorectal, head and neck)	NCT00761644, NCT00877773, NCT01054313, NCT00610493, NCT00726583 (phases I/II)	140 (217)	*PIK3CA* mutation	NA	Coexisting *KRAS* mutation (tissue-specific)	Janku et al. (2011, 2012)
Everolimus (mTOR), single agent	Colorectal, breast, melanoma, pancreas, HNSCC	ND (retrospective subgroup analysis; phase I/II)	43	*PIK3CA* mutation PTEN loss of function	NA	Coexisting *KRAS* mutation	Di Nicolantonio et al. (2010)
Temsirolimus (mTOR), combined	Ovarian, uterine, cervix, breast cancer	NCT00761644 (phase I)	74	*PIK3CA* mutation PTEN loss of function	NA	NA	Moroney et al. (2011)
Everolimus (mTOR), combined	Neuroendocrine carcinoma	NCT00113360 (retrospective subgroup analysis; phase II)	60 (17 assessed)	NA	p-AKT (Thr308; PSF)	NA	Meric-Bernstam et al. (2012)
Everolimus (mTOR), single agent	NSCLC	NCT00124280 (phase I)	58 (40 assessed)	NA	p-AKT (Ser473, Thr308; PFS)	NA	Soria et al. (2009)
Everolimus (mTOR), single agent	SCLC	NCT00374140 (phase II)	40 (22 assessed)	NA	S6K	NA	Tarhini et al. (2010)
Temsirolimus (mTOR), single agent	Glioblastoma multiforme	NCT00016328 (phase II)	56 (43 assessed)	NA	p-S6K (Thr421/Ser424)	NA	Galanis et al. (2005)
Deforolimus (Ridaforolimus; mTOR), single agent, and combination	Sarcoma	NCT00288431 NCT00093080 (phase I/II; retrospective subgroup analysis)	20	NA	p-rpS6 (Ser235/236)	NA	Iwenofu et al. (2008)
Temsirolimus (mTOR), single agent	Neuroendocrine carcinoma	NCT00093782 (phase II)	37 (35 assessed)	NA	p-mTOR (Ser2448)	NA	Duran et al. (2006)
Everolimus (mTOR), single agent	Breast cancer	NCT00255788 (phase II)	49 (47 assessed)	NA	None (PTEN, p-AKT, CA9, ER, PR, HER2 assessed)	NA	Ellard et al. (2009)
Ridaforolimus (mTOR), single agent	Bone and soft tissue sarcoma	NCT00093080 (phase II)	212 (∼80 assessed)	NA	None (p27Kip1, IGF-1R, PTEN, p-S6K, 4E-PB1, eIF4E, p-AKT, FKBP12)	NA	Chawla et al. (2012)
Temsirolimus (mTOR), single agent	Epithelial ovarian and peritoneal malignancies	NCT00429793 (phase II)	54 (51 assessed)	NA	None (p-AKT, p-mTOR, p-S6K, p-4E-BP1, cyclin D1)	NA	Behbakht et al. (2011)

*CA9, carbonic anhydrase IX; HNSCC, head and neck squamous cell carcinoma; NA, not assessed; ND, not defined; NSCLC, non-small cell lung cancer; PFS, progression-free survival; SCLC, small cell lung cancer*.

The vast majority of completed to date trials testing PI3K pathway inhibitors in tumor types other than renal cell carcinoma also focused on rapalogs, but only few studies assessed potential genomic predictors (Table 3). Based on the rationale that *PIK3CA* mutations may predict response to PI3K pathway inhibitors, breast, cervical, endometrial, and ovarian cancers were sequenced for the presence of activating *PIK3CA* mutations and treated with different allosteric mTOR inhibitors (i.e., rapalogs) or the PI3K inhibitor PX866 either as single agent or combination in a prospective phase I clinical trial. A partial response was observed in 30% of the 23 patients with tumors harboring a *PIK3CA* mutation in contrast to 10% of 70 patients whose tumors were *PIK3CA* wild-type (Janku et al., 2012), consistent with the preclinical observations. Interestingly, whilst in preclinical models mutations in *KRAS* have been found to confer resistance to PI3K pathway inhibition, as discussed above, in this trial 2/7 ovarian cancer patients with coexisting *PIK3CA* and *KRAS* or *BRAF* mutations responded to the anti-PI3K pathway treatment (Janku et al., 2012). This finding may be tumor type-specific, given that the same group had previously described that colorectal cancers harboring simultaneous *PIK3CA* and *KRAS* mutations were resistant to PI3K pathway inhibitor treatment (Janku et al., 2011). A similar, but not statistically significant, trend was observed in a retrospective subgroup analysis of 43 patients with different tumor types but most frequently colorectal cancer from phase I/II clinical study of single agent Everolimus (Tabernero et al., 2008; Di Nicolantonio et al., 2010). Patients whose tumors harbored *PIK3CA* mutations or PTEN loss of function were more likely to benefit from Everolimus, except in presence of coexistent *KRAS*/*BRAF* mutations (Di Nicolantonio et al., 2010). A high percentage of responders with PI3K pathway aberrations as determined by *PIK3CA* mutations or PTEN loss of function was also reported in a phase I trial of liposomal Doxorubicin, Bevacizumab, and Temsirolimus (Moroney et al., 2011). Taken together, these results suggest *PI3KCA* gain-of-function mutations may predict sensitivity to PI3K pathway inhibitors, whereas *KRAS* and *BRAF* mutations may lead to resistance in some tumor types such as colorectal cancer. Importantly, however, the data available demonstrate that *PIK3CA* activating mutations are neither required nor sufficient for a tumor to be sensitive to PI3K pathway inhibitors, and that a substantial proportion of cases with *PIK3CA* activating mutations may be *de novo* resistant to these agents.

Not only genomic predictors but also PI3K pathway activation state as determined by expression levels of markers upstream of mTORC1, such as p-AKT, or downstream of mTORC1, such as p-S6K (Figure 1), have been shown to correlate with sensitivity to allosteric mTOR inhibitors in breast cancer cell lines *in vitro* (Noh et al., 2004; Meric-Bernstam et al., 2012). In fact, several clinical trials testing rapalogs evaluated PI3K signaling biomarkers on baseline tumor tissue by IHC rather than performing sequencing analysis. In a phase II study, high p-AKT levels on baseline and on-treatment fine needle aspirations of tumors from patients with neuroendocrine carcinoma (*n* = 17) assessed by reverse phase protein arrays correlated with longer progression-free survival (PFS; Meric-Bernstam et al., 2012). Moreover, in NSCLC patients treated with Everolimus (*n* = 40), p-AKT levels at baseline determined by IHC were reported to be independent predictors of PFS (Soria et al., 2009; Table 3). It should be noted, however, that the authors emphasized that tissue fixation had large effects on immunoreactivity when assessing phosphorylated proteins, which may compound the implementation of this IHC predictive test in clinical practice.

In a phase II trial, p-S6K levels assessed by IHC in baseline glioblastoma multiforme samples were associated with single agent Temsirolimus response (*n* = 44; Galanis et al., 2005). Also in a small retrospective subgroup analysis of two phase I/II clinical trials p-rpS6, downstream of p-S6K, was correlated with early response of sarcomas to the rapalog Deforolimus (i.e., Ridaforolimus) alone or in combination with doxorubicin (*n* = 20; Iwenofu et al., 2008). Not only expression levels of activated (i.e., phosphorylated) S6K/rpS6 have been found to correlate with response to allosteric mTOR inhibitors, but in phase II study, total S6K expression in baseline SCLC tumor tissue defined by IHC was reported as a potential predictive biomarker for the therapeutic benefit of Everolimus (*n* = 22; Tarhini et al., 2010). In addition, higher baseline levels of p-mTOR itself assessed by IHC predicted for a better response to Temsirolimus in patients with neuroendocrine carcinoma in a phase II study (*n* = 35; Duran et al., 2006).

Other, similarly powered phase II trials however did not identify any correlates between potential biomarkers assessed in archival tumor material using IHC and treatment response (Table 3). In metastatic breast cancers, no association between p-AKT, PTEN, CA9, ER, progesterone receptor or HER2 expression, and response to Everolimus was found (Ellard et al., 2009). In bone and soft tissue sarcomas, an extended subgroup analysis (*n* ≈ 80; Chawla et al., 2012) did not confirm the previously published analysis on 20 patients, which identified p-rpS6 levels as Ridaforolimus response predictors (Iwenofu et al., 2008). In fact, neither the potential biomarkers upstream of mTORC1, including PTEN, p-AKT, FKBP21, or IGF-1R, nor downstream of mTORC1, p-S6K, 4E-BP1, eIF4E, or p27kip1, were predictive of clinical benefit response to Ridaforolimus (Chawla et al., 2012). Furthermore, p-AKT, p-mTOR, p-4E-BP1, and cyclin D1 expression levels in epithelial ovarian and peritoneal tumors were shown not to be associated with partial/complete tumor responses to Temsirolimus, however cyclin D1 expression seemed to correlate with PFS ≥6 months (Behbakht et al., 2011).

The completed phase I/II clinical trials to date are to some extent consistent with the preclinical observations in that tumors harboring *PIK3CA* mutations may be more likely to respond to PI3K pathway inhibitors. It is important to note, however, that not all patients with tumors harboring *PIK3CA* mutations are sensitive to PI3K pathway inhibitor treatment, and, on the other hand, that also subsets of patients with wild-type *PIK3CA*/*PTEN* cancers are responsive. The results from studies analyzing the activation state of PI3K pathway components by IHC are variable and no consistent determinant of response has been identified to date.

## Development of Molecular Tools for the Stratification of Cancer Patients

For the development of molecular markers for patient stratification in clinical trials testing the efficacy of PI3K pathway inhibitors, it is crucial to take into account the observations that in some tumor types, either *PIK3CA* activating mutations or PTEN loss of function are predictors of sensitivity, whereas in other tumor types, both predict sensitivity to these agents (Table 2). These data imply that the mutational repertoire and the epistatic interactions between different components of the PI3K pathway may be distinct in different tumor types, that genetic lesions in different components of the pathway may not have the same functional effects in different tumor types, and that a genetic determinant identified in one cancer type may not necessarily be applicable to another. This is perhaps best exemplified by *BRAF*^V600E^ mutations, which are predictive of response to Vemurafenib in melanoma, however colorectal cancer patients harboring oncogenic *BRAF*^V600E^ mutations derive limited if any benefit from this drug due to increased EGFR expression (Prahallad et al., 2012). Likewise, the clinical trials discussed above provide evidence to suggest that ovarian cancers with coexisting *PIK3CA* and MAPK pathway mutations may be sensitive to PI3K pathway inhibition, whereas colorectal cancers harboring the same repertoire of mutations affecting these genes may be resistant (Di Nicolantonio et al., 2010; Janku et al., 2011, 2012).

Results from preclinical studies performed have further suggested that cancer cells harboring *PIK3CA* mutations might be among the most sensitive to single agent PI3K pathway inhibitors. These data were in part confirmed in the three clinical trials assessing tumor *PIK3CA* mutational status (Di Nicolantonio et al., 2010; Janku et al., 2011, 2012; Moroney et al., 2011). The predictive value of the PTEN status is however less clear, as in some clinical trials an association between PTEN deficiency and PI3K pathway inhibitor response was found (Di Nicolantonio et al., 2010; Moroney et al., 2011) but not in others (Ellard et al., 2009; Figlin et al., 2009; Chawla et al., 2012). A similar picture is seen when expression levels of phosphorylated proteins of the PI3K pathway are used as a read-out of its activation state and determinant of response (Table 3). IHC of phosphorylated proteins has proven challenging (Soria et al., 2009; Pinhel et al., 2010) and also PTEN staining is not routinely performed. Recent reports focused on the reproducibility of PTEN staining protocols and scoring (Sakr et al., 2010; Garg et al., 2012), however guidelines for accurate PTEN testing and its utility as predictive marker have yet to be established.

Early clinical trials often analyze archival tissue of the primary tumor for the presence of specific mutations and the response of the metastatic lesions are correlated with the mutational status. Recent analyses of paired primary tumors and metastases have revealed that there is a high level of discordance in PTEN expression level and *PIK3CA* mutation status, which may influence patient selection and response to PI3K targeted therapies (Dupont Jensen et al., 2011; Gonzalez-Angulo et al., 2011).

Despite the interest in the development of biomarkers for patient selection in clinical trials testing PI3K pathway inhibitors, none of the biomarkers tested so far is supported by level I evidence. Importantly, however, one of the most exciting results of allosteric mTOR inhibitors in the context of a clinical study was the BOLERO-2 trial, where patients with ER-positive advanced breast cancers resistant to aromatase inhibitors were randomized to receive Exemestane (a non-steroidal aromatase inhibitor) plus Everolimus or Exemestane plus Placebo (Baselga et al., 2012). The rationale for this stemmed from preclinical observations that resistance to endocrine therapy in breast cancer is associated with activation of the PI3K pathway (Miller et al., 2011). Despite the lack of a patient stratification biomarker, this trial demonstrated that addition of Everolimus to Exemestane increased the median PFS from 4.1 to 10.6 months (Baselga et al., 2012). Although a substantial proportion of patients included in this trial may harbor *PIK3CA* activating mutations, given that they are more likely to occur in ER-positive postmenopausal patients (Kalinsky et al., 2009), other mechanisms resulting in PI3K pathway activation are likely to play a role in resistance to endocrine therapy. The material from this trial will constitute a unique resource to determine the genomic and epigenomic determinants of sensitivity to concurrent mTOR inhibition and endocrine treatment in breast cancer.

## Future Perspectives and Challenges

Despite the critical role of the PI3K pathway in cancer, the introduction of single-agent PI3K pathway inhibitors into the clinic may be challenging. In fact, of all PI3K pathway inhibitors discussed here, one of the most exciting targeted agents is the p110δ inhibitor CAL-101, which has shown remarkable clinical activity in certain hematological diseases including chronic lymphocytic leukemia. Inhibition of p110δ is though to target both the malignant B cells and the tumor microenvironment of chronic lymphoid leukemia (Fruman and Rommel, 2011). The clinical trials performed thus far using allosteric mTOR inhibitors as single agents have seen some stable diseases and partial responses, however by no means are these responses as dramatic as for example for Vemurafenib in *BRAF* mutant melanoma (Flaherty et al., 2010). Based in the preclinical data, kinase inhibitors seem to target the PI3K pathway more robustly and, in contrast to allosteric mTOR inhibitors, also promote apoptotic effects *in vitro* and *in vivo* (Brachmann et al., 2009; O’Brien et al., 2010; Weigelt et al., 2011). Several feedback loops upon PI3K/AKT/mTOR inhibition have been described, which amongst others lead to activation of the MAPK signaling pathway or re-activation of the PI3K pathway (reviewed in Carracedo and Pandolfi, 2008; Efeyan and Sabatini, 2010; Chandarlapaty, 2012; Laplante and Sabatini, 2012). These feedbacks may play a role in the modest responses observed thus far using single agent rapalogs, and for the optimal activity of PI3K pathway inhibitors, co-administration with other agents may be required.

Numerous clinical trials are currently testing the safety and efficacy of combination PI3K pathway and MEK inhibitors in advanced solid tumors to target both the driver and potential “escape” pathways. As discussed above, such combinatorial approach has been successfully performed in breast cancer, where the combination of an aromatase inhibitor with Everolimus led to substantial improvement of PFS (Baselga et al., 2012). For the identification of optimal combinations of PI3K pathway inhibitors with other agents, *Drosophila* models may provide an effective tool as these have been successfully employed for the identification of agents with optimized pharmacological profiles (Das and Cagan, 2010; Dar et al., 2012). For combinatorial treatments it will be crucial to understand the activation of negative feedback loops in the PI3K pathway and the cross-talk with other pathway upon inhibition of its different components, and whether the feedback activation is dependent on specific epistatic interactions and distinct in tumors from different anatomical sites.

Drug sensitivity and resistance are likely to constitute convergent phenotypes, meaning that they may be driven by distinct genetic aberrations in the same tumor type (Gerlinger and Swanton, 2010; Turner and Reis-Filho, 2012; Weigelt et al., 2012; Yap et al., 2012). It has become apparent from *in vitro* studies that there are significant correlations between specific mutations and treatment response, however the negative predictive value of these mutations is often poor and not all sensitive cancers are identified by single mutations/single gene panels. For example, O’Brien et al. (2010) showed that in their cell line panel tested, *PIK3CA* mutations and *HER2* amplification showed excellent specificity (100 and 95%, respectively) and a high positive predictive value, but relatively low sensitivity (∼30%) and a poor negative predictive value as single markers in predicting drug responsiveness in the cell line panel analyzed. Additional biomarkers will therefore be required to identify all patients likely to respond to PI3K pathway inhibitors. To date, the majority of studies have focused on the analysis of *PIK3CA* mutations or PTEN deficiency as potential determinants of PI3K pathway inhibitor response. However, also activating mutations in other components of the pathway, such as *PIK3R1* (Jaiswal et al., 2009; Urick et al., 2011) or *mTOR* (Sato et al., 2010; Hardt et al., 2011), or loss of function of TSC1/2 (COSMIC; El-Hashemite et al., 2003; Sjodahl et al., 2011) or INPP4B (Gewinner et al., 2009; Fedele et al., 2010) may play a role in PI3K pathway inhibitor response. Furthermore, and as mentioned above, the remarkable single agent activity of the PI3K isoform specific p110δ inhibitor CAL-101 in chronic lymphocytic leukemia, a disease in which PI3K pathway aberrations are relatively rare, emphasizes that some targeted agents may be effective *in vivo* due to targeting of tumor microenvironment interactions (Fruman and Rommel, 2011), which are unlikely to be uncovered using conventional *in vitro* cell culture models or by the genomic characterization of tumor cells only.

With the advent of massively parallel sequencing technologies, several studies have documented intra-tumor genetic heterogeneity in solid cancers (reviewed in Turner and Reis-Filho, 2012; Yap et al., 2012), and revealed that certain mutations, including *PIK3CA* or *PTEN* mutations, may be only prevalent in a subset of tumor cells in a given cancer (Gerlinger et al., 2012; Shah et al., 2012). This has not only consequences for cancer drug resistance and the clinical utility of single agent targeted therapy, but also questions whether potential biomarkers assessed in a single biopsy will be representative of the entire tumor.

Given the crucial role of the PI3K pathway in cancer, inhibitors of its components are expected to be effective in subsets of many different cancer types. Preclinical models have proven useful in the identification of potential predictive biomarkers, however tissue collection and assessment of biomarkers even in early clinical trials are crucial, as is the development of robust and accurate companion diagnostics. With the number of ongoing clinical trials currently testing a wide gamut of PI3K pathway inhibitors, our community should expect a wealth of data, which will help improve therapeutic strategies for cancer patients.

## Glossary

### Phosphoinositide 3-kinase classes

According to their structures and substrate specificities, PI3Ks are divided into three classes, and class I PI3Ks are directly activated by cell surface receptors (Liu et al., 2009). Class IA PI3Ks are heterodimeric lipid kinases composed of a p110 catalytic subunit (isoforms p110α, p110β, and p110δ, encoded by *PIK3CA*, *PIK3CB*, and *PIK3CD*, respectively), and a regulatory subunit (p85α and its splice variants p55α and p50α), p85β, and p55γ, encoded by *PIK3R1*, *PIK3R2*, and *PIK3R3*, respectively); the class IB PI3K is composed of the p110γ catalytic subunit, encoded by *PIK3CG*, and the regulatory subunit p101, p84/p87 (Liu et al., 2009; Vanhaesebroeck et al., 2010).

### Rapamycin and rapamycin analogs (“rapalogs”)

Mechanistic target of rapamycin is a serine/threonine kinase that interacts with several proteins to form two distinct signaling complexes called mTORC1 and mTORC2 (Laplante and Sabatini, 2012). Rapamycin and rapamycin analogs (“rapalogs”) bind the FK506-binding protein (FKBP12) and together target preferentially the mTORC1 by an allosteric mechanism, however prolonged treatment may also inhibit mTORC2 and disrupt its main substrate AKT, possibly in a tissue-specific manner (Sarbassov et al., 2006; Lamming et al., 2012).

## Conflict of Interest Statement

The authors declare that the research was conducted in the absence of any commercial or financial relationships that could be construed as a potential conflict of interest.
